# Prospecting movements link phenotypic traits to female annual potential fitness in a nocturnal predator

**DOI:** 10.1038/s41598-023-32255-7

**Published:** 2023-03-28

**Authors:** Paolo Becciu, Robin Séchaud, Kim Schalcher, Céline Plancherel, Alexandre Roulin

**Affiliations:** 1 Department of Ecology and Evolution, University of Lausanne, Lausanne, Switzerland; 2 Agroecology and Environment, Agroscope, Reckenholzstrasse 191, 8046 Zurich, Switzerland

**Keywords:** Ecology, Evolution, Zoology

## Abstract

Recent biologging technology reveals hidden life and breeding strategies of nocturnal animals. Combining animal movement patterns with individual characteristics and landscape features can uncover meaningful behaviours that directly influence fitness. Consequently, defining the proximate mechanisms and adaptive value of the identified behaviours is of paramount importance. Breeding female barn owls (*Tyto alba*), a colour-polymorphic species, recurrently visit other nest boxes at night. We described and quantified this behaviour for the first time, linking it with possible drivers, and individual fitness. We GPS-equipped 178 female barn owls and 122 male partners from 2016 to 2020 in western Switzerland during the chick rearing phase. We observed that 111 (65%) of the tracked breeding females were (re)visiting nest boxes while still carrying out their first brood. We modelled their prospecting parameters as a function of brood-, individual- and partner-related variables and found that female feather eumelanism predicted the emergence of prospecting behaviour (less melanic females are usually prospecting). More importantly we found that increasing male parental investment (e.g., feeding rate) increased female prospecting efforts. Ultimately, females would (re)visit a nest more often if they had used it in the past and were more likely to lay a second clutch afterwards, consequently having higher annual fecundity than non-prospecting females. Despite these apparent immediate benefits, they did not fledge more chicks. Through biologging and long-term field monitoring, we highlight how phenotypic traits (melanism and parental investment) can be related to movement patterns and the annual potential reproductive output (fecundity) of female barn owls.

## Introduction

Biologging is crucial for understanding patterns of movement-related animal behaviour and how they are directly linked to the dynamics and persistence of populations. This technology is allowing for the discovery, quantification, and analysis of many previously unknown or unquantifiable behaviours^1,2^. Nowadays, with the accumulation of large quantities of biologging data we can finally answer long-standing questions in behavioural and movement ecology, such as how the internal state (including individual phenotypic characteristics), social interactions and abiotic environment can affect movement patterns and consequently individual fitness^3–5^. Recently, studies focused on how certain phenotypic characteristics or internal states could affect movement-related behaviours^6^, or how the latter would affect individual fitness and/or survival^7–9^, but rarely have studies explored the link between individual phenotype, its interactions with the environment, and consequent fitness output^10,11^.

Movement-related behaviours with clear trajectorial patterns are currently often recognisable as related to foraging^12,13^, resource searching^14^, and commuting^15,16^, but in other circumstances movement patterns require more attention and a better understanding of the social and environmental contexts^5^. A deeper study of movement patterns paired with environmental context-related information can help in hypothesising possible causes, mechanisms, and consequences of a newly identified behaviour^5,17,18^. Recently, post-tracking analytical tools are easing this process, helping to recognise and find repeated and consistent movement-related patterns across a large pool of individuals in order to identify a behaviour^19–21^. Identifying and analysing recursive movement patterns, such as repeated visits to specific locations like foraging patches, nests, or dens, is a powerful way to detect behavioural structure in movement trajectories^19,20,22^.

In general, visiting potential nest sites one or multiple times can be highlighted by recursive movement and will probably be associated with sampling spatial, resource and social information to collect knowledge for future breeding-site-choice decisions^23–27^. If not too expensive in terms of time and energy, acquiring important information related to the breeding site is expected to enhance individual fitness^28,29^. Usually, birds prospect for nesting sites in the pre-breeding period^23,24^, but it is not rare to find post-breeding prospectors^30–32^. In addition, previous tracking studies on prospecting behaviour focussed on larger scale prospecting (e.g., colonies and possible breeding areas)^27,33^, often triggered by failed reproductive attempts^34,35^, mostly in seabird species. Furthermore, it is rarely reported for single- and multiple-brooded species to have individuals prospecting while breeding, specifically towards the end of chick rearing (but see^36^). While breeding, the individual prospector could be also influenced by, or associated with, the partner’s behaviour, given that the reproductive system is based on a certain degree of cooperation within the pair. Unfortunately, studies with simultaneously tracked pairs are uncommon^37^ and none have investigated this topic to our knowledge. Although not in birds, a relationship between males and female movement patterns was previously found in a social mammal species, where dispersing and prospecting other sites was driven by food competition and territorial males^38^.

In this study, we present the (re)visiting of surrounding nest-boxes as a possible prospecting behaviour of breeding female barn owls (*Tyto alba*) in order to understand possible triggers and fitness-related consequences. We could quantify this prospecting behaviour from GPS-equipped wild barn owl breeding pairs in western Switzerland over five years in an intensive agricultural area^9,39^. Combining the high-frequency sampling rate with a behavioural classification method and recursive analysis we were able to characterize barn owl prospecting behaviour including visits to other nesting sites and time spent there^19,39^. Barn owls are a colour-polymorphic species breeding in nest boxes, with males more involved than females in chick provisioning and with both males and females often raising two broods per season^40,41^. Numerous female individuals were visiting other nest sites during their chick-rearing phase. Performing such behaviour could redirect time and energy from chick rearing to an activity of which we do not know the benefits and related costs.

We expected that female prospecting behaviour to be related to (a) variables related to the ongoing clutch (i.e., brood size, chicks growth rate, laying date, etc.) (b) individual factors (i.e., age, body condition, melanism, etc.), and (c) partner characteristics (i.e., male's age, home range size, chick’s provisioning rate, etc.). With (a) testing for potential fitness effects, while (b) and (c) testing for phenotype traits. Each of these variables could reasonably cause a long- or short-term behavioural change that would translate into different movement patterns (e.g., onset and amount of prospecting for nest boxes). In addition, we predict that (d) previous knowledge of the surrounding nesting boxes (e.g., prospecting female already nested there) and their success history (e.g., often occupied in previous years) could be predicting the probability of a nest box to be visited^26,31^. We also expected that (e) prospecting behaviour predicts the possibility of re-nesting and starting a second clutch^41^. Finally, (f) performing this behaviour could predict an increase in female fecundity (if prediction ‘e’ is met), but not necessarily a higher number of fledglings or better-quality fledglings^41,42^. Female fecundity (annual number of eggs produced) is likely increased when a second clutch is laid, but their overall fitness (number of fledglings) heavily depends on the ability of their male partner to provide food to the chicks.

Our study shows the mechanisms of different breeding strategies in a cosmopolitan and colour-polymorphic species, such as the barn owl, with deep implications to its individual potential fitness. Furthermore, this is the first evidence of breeding female prospecting (at specific nesting locations) using high-resolution simultaneous pair tracking in a cosmopolitan nocturnal raptor.

## Materials and methods

### Study area and barn owl population monitoring

The study was carried out between 2016 and 2020 in an intensive agricultural landscape in western Switzerland where a wild population of barn owls breeds in nest boxes attached to barns^43^. For the first 2 weeks after hatching, the adult females stay almost entirely in the nest providing warmth to their offspring and distribute food brought by the male. After this period, both parents hunt small mammals, with the male being the main contributor to the chick provisioning^9,40^. Data relative to breeding biology such as laying dates, number of eggs, nestlings and fledglings, body measurements, plumage pheomelanism (colour) and eumelanism (spottiness) assessment were taken as part of annual monitoring of the species for over 30 years^41^.

### GPS tags, deployment, behaviour annotation and home range size

178 female barn owls and 122 male partners were tagged using GiPSy-5 GPS tags (2016–2017) and Axy-Trek GPS and accelerometer tags (2018–2020) (https://www.technosmart.eu/, Technosmart, Italy). Each tag collected the location and the date-time every 10 s (GiPSy-5) or every second (Axy-Trek), from 30 min before dusk until 30 min after dawn, covering the entire owl nocturnal activity period. Breeding barn owls were captured at their nest site when the oldest offspring was 25 days-old on average (SD = 2.8), equipped with GPS tags and released at the capture site. Movement data was processed for behaviour annotation and calculation of home range size following Séchaud et al. (2021, 2022). We resampled every 10 s the higher resolution tracks at 1 s (Axy-Trek), to avoid bias in estimating parameters concerning visits to nest boxes. More details about GPS deployment, behavioural categorization and calculation of home range size are reported in the Electronic Supplementary Materials. Data handling, calculation of parameters, variables and statistical analyses were performed in the statistical environment R 4.0.2^44^ with RStudio^45^ as graphic user interface.

### Identification of prospecting behaviour

We quantified the recursive movement of 171 female barn owls (excluding individuals with less than 3 nights of tracking) at nest boxes around their own with the “recurse” R package^19^, using the function *getRecursionsAtLocations*. This function calculates number of visits at specific given locations with the option to include a buffer radius and a residence time threshold. We applied a circular buffer of 20 m around nest boxes to control for GPS errors and two temporal thresholds: (1) 1 min to have high resolution data on their time spent close to the visited nest using *calculateIntervalResidenceTime* (calculating the residence time during user-specified intervals in the radius around each location), and (2) 600 min to count the number of different locations (nest boxes) visited per night. With the first threshold we aimed at calculating time duration of every possible visit to the nest box with a time buffer between repeated visits of 1 min. The second threshold was used as a qualitative measurement of which nest box was visited per tracking night (hence the time buffer of 10 h). From the initial ‘recurse’ object data, we calculated a series of parameters per night and per individual: overall visits to other nest boxes, number of revisits per nest box, number of nest boxes visited, median and sum time spent at the nest boxes. These parameters gave us an idea of the different expressions of the prospecting behaviour. For example, some females have many revisits but only to few nest boxes, while others visit many different nest boxes but only one time per box. We also created a binomial variable, called “prospecting [0, 1]”, to simplify the behaviour. If a female visited at least a nest site other than her own it would account for 1, else 0. In our analyses, we used four response variables:

- prospecting [0, 1]: if the behaviour was expressed at least once [1] or never [0];
- mean visits per night: number of total visits to nest boxes divided by the number of nights the female was tracked for, giving an idea of the motivation to (re)visit, regardless of the number of nest sites (re)visited;
- nest boxes visited: number of nest boxes visited at least once, to account for the diversity of places visited;
- median time (minutes) at nest sites per night: this parameter relates to the information acquisition of the nest box visited.

**Figure 1:**
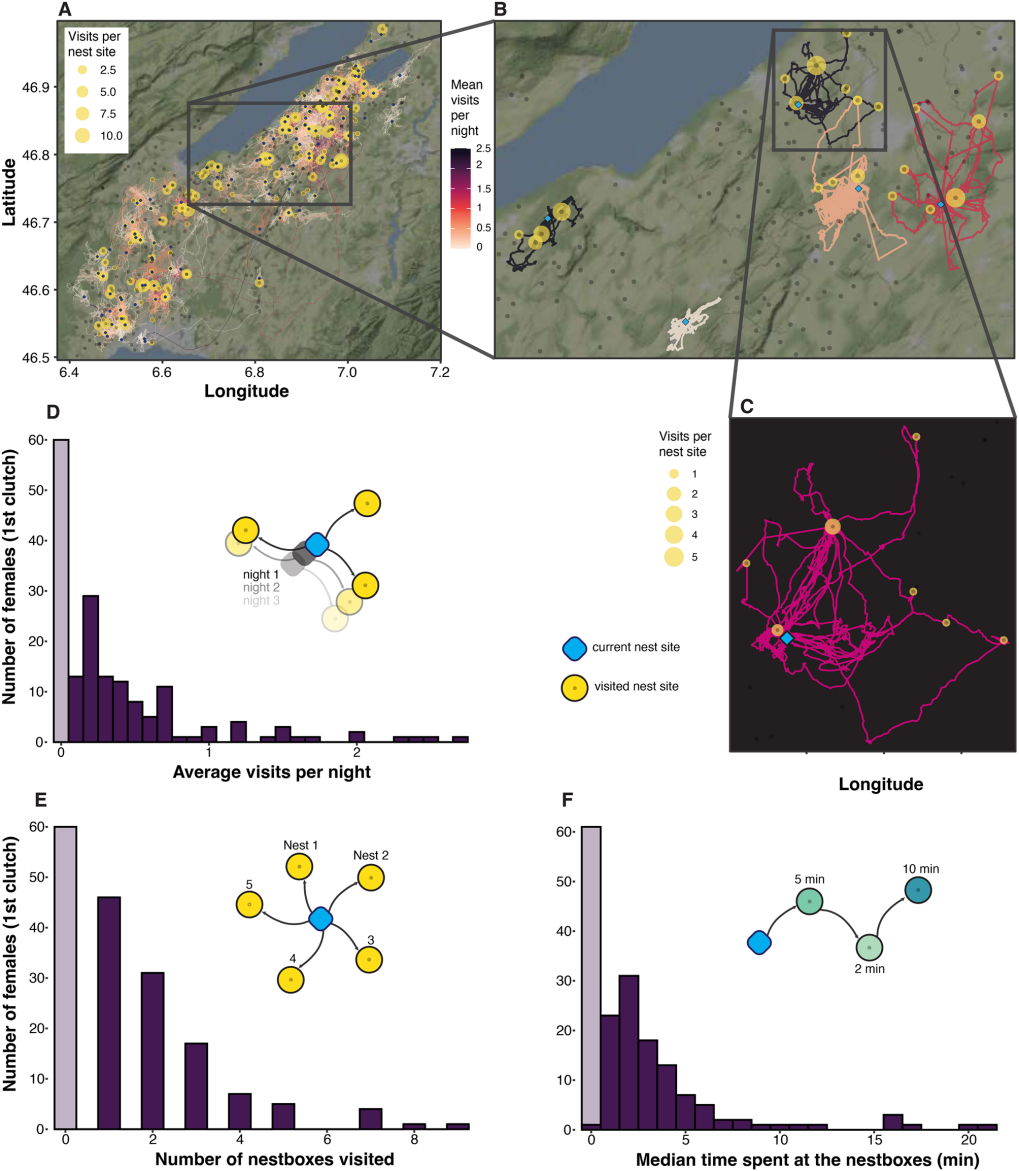
Schematic description and visualization of prospecting behaviour expressed by female barn owls. (**A**) Map of the study area with all tracked females considered for analysis (n = 171), highlighting average revisits per night (colour of the trajectories) and the number of visits per nest boxes (yellow circles). (**B**) Example of five female tracks with different degrees of prospecting behaviour or none (the lightest coloured track). (**C**) One track in detail, the colour of this track is for visualization purposes. (**D–F**) Histograms of the different parameters chosen to analyse the prospecting behaviour: in transparent violet females that did not visit any nest site except their own (n = 60), in dark violet the variation of the different prospecting parameters expressed by the females that visit at least one nest (n = 111).

For the continuous response variables (mean visits per night, nest sites visited, median time at nest sites per night) we also created a subset of the data excluding the non-prospecting females (prospecting = 0), in order to analyse variation among the prospecting females (Fig. 1).

### Explanatory variables

We divided the predictors into groups that match the expectations (a), (b) (c), (d), (e) and (f) in the “Introduction”:

- We also considered a group of variables relative to the ongoing breeding situation (during the tagging period). “Brood size” at the beginning of tag deployment as well as the loss of nestlings within the tagging period (“Brood size diff.”) as proxies of pressure on parental food provisioning duties. We also considered the "Wing growth rate” of the oldest chick in the brood during the tagging period to have an estimate of parental investment. “Laying date” (Julian date, as integer starting 1st of January of every year) was also included to account for when in the breeding season they laid the first clutch eggs, usually the earlier the better for a second re-nesting chance in the same season^41^.
- Female characteristics that might influence their prospecting behaviour expression were considered. “Daily mass variation”, defined as the difference in body mass between tagging and tag retrieval divided by the number of days tag were deployed. “Body condition” as residuals of a linear regression between wing length and body mass^46^ recorded at the time of tagging. “Age” as factor (1st-year female or older than 1 year—since the exact year of birth is not available for all individuals). Eumelanin expressed as number of spots (“Spottiness”) per unit of space (count of spots in a 60 × 40 mm frame) and as spot diameter on breast and belly, these factors can be related to different behavioural and breeding aspects in female barn owls^47–49^. Pheomelanin as breast and belly mean colour (“Colour”) indexed from white (value of 8) to reddish-brown (value of 1), this trait was also found to be related with hunting behaviour and environmental factors^11,50,51^.
- In addition, we considered a set of variable relative to the female’s partner characteristics and behaviour. “Body condition”, “Age”, “Spottiness” and “Colour” as described above for the female parameters. We also added components of male hunting and movement behaviour such as “Home range size”^9^, "Provisioning rate” per night defined as visits to their own nest by the males (see Click or tap here to enter text., and proportion of time they spent perching (“Time spent perching”), and therefore resting, at night as a proxy for their activity rate^39^.
- The probability of visiting a specific nest box multiple times was placed in relationship to variables relative to the nest boxes themselves, such as: proportion of nest box usage in the previous 15 years (“Nest active previous years”), binomial value of nest activity in the current year (“Nest active current year”), number of nest boxes active or not in a radius of 3.3 km around the current nest (“Nest density”, radius corresponding to the 3^rd^ quartile of the visited nest boxes distances), distance (log-transformed) between breeding nest and visited nest boxes (“Nest distance”), number of breeding attempts by the female at visited nest boxes in the previous 15 years (“Breeding attempts previous years”), and number of fledglings produced by any barn owl pair at visited nest boxes in the past 15 years (“Mean fledglings previous years”), as a proxy for long-term nest box quality.
- The explanatory variables used for predicting the probability of having a second clutch by the tracked female barn owls were “Laying date” (for the aforementioned reason), “Brood size” to account for the effort made in the first clutch, “Home range size” of their partner in the first clutch as proxy of their quality^9^, “Age” of the female, final reproductive success of the first clutch expressed as fledglings produced divided the number of eggs laid (“Fledglings to nestling ratio”). In addition, we included one of the four prospecting parameters after a variable selection among them (explained below).
- Annual sum of eggs and fledglings were related to prospecting parameters as explanatory variables.

Prior any statistical analyses we checked for correlations between the prospecting parameters and the number of available nest boxes in a range of 3.3, 5, 10, 20 km radius around the nest site of the tracked female. We did not find any strong correlation at any spatial scale hence we assumed that prospecting behaviour was not related to the availability of surrounding nest boxes (see Electronic Supplementary Materials). Also, female prospecting tracks are relative to their first clutch breeding event (i.e., tracked females carrying a second clutch were excluded).

### Data analysis

In order to test our first three predictions (a), (b) and (c), concerning possible effects of partner-, brood- and female-related variables on the four prospecting parameters (“prospecting[0,1]”, “mean visits per night”, “nest sites visited”, “median time at nest sites per night”), we created different subsets per prediction since the amount of missing data would differ between the sets of explanatory variables. Therefore, we built a series of (generalised) linear mixed models (GLMMs or LMMs) with different error distributions depending on the response variable: binomial for “prospecting[0, 1]”, Tweedie distribution for “mean visits per night” and “median time at nest sites per night”, Negative Binomial with quadratic parameterization for “nest sites visited”, Gaussian in models without non-prospecting females. These distributions were chosen to deal with zero inflation and overdispersion in the data. The combination between the four response variables and the three sets of predictors resulted in 21 (G)LMMs. To avoid multicollinearity issues, we chose the most biologically meaningful variable from pairwise Spearman rank correlation |ρ|> 0.6. This ensured that all the predictors in the GLMMs had a VIF (variance inflation factor) < 3^52^. For each of the 21 global models, we proceeded to select the optimal structure of the fixed component using a multi-model selection framework ranking the selected models according to the Akaike information criterion^53^, using an automated stepwise model selection procedure in which models are fitted through repeated evaluation of modified calls extracted from the model containing all the meaningful variables, corrected for small sample sizes (AIC_c_)^54^. Since no candidate model had a weight greater than 70%, parameters were estimated as the weighted averages of the set of models that represented 95% confidence weight^55^ by averaging by the zero method^56^. To distinguish effects of predictors on response variables that differed from zero, 95% confidence intervals (C.I.) for each predictor were calculated. Furthermore, we used the Akaike weights (w_i_)^57^ to assess the relative importance of the different variables (sum of Akaike weights, Σw_i_).

To test if previous knowledge of surrounding nest sites could predict the probability of multiple visits to a specific nest site—prediction (d)—we ran a binomial GLMM with a response variable reflecting if a nest site was visited only once [0] or more than once [1].

To test prediction (e) that prospecting behaviour could possibly predict the probability of re-nesting (having a second clutch), we first set up a series of univariate binomial GLMMs using as binomial response variable if the female nested once or twice in the season (0 = one clutch, 1 = two clutches per year). We included as predictors the four prospecting parameters and then compared the four univariate models to choose the best predictor (model with lowest AIC_c_). Using the best prospecting variable, we then built a more inclusive model including variables that could meaningfully predict re-nesting behaviour (as explained in the previous section). We followed the multi-model inference method described above to evaluate the most important predictors and their relationship with the probability of re-nesting a second time in prospecting and non-prospecting female barn owls.

Finally, we tested possible long-term effects of prospecting behaviour—prediction (f)—evaluating the best prospecting predictor influencing the annual sum of eggs and fledglings by comparing univariate models as described above. We did not build a global model because the forces acting on annual fecundity and fitness are multiple, and our simplification is a starting point connecting the prospecting behaviour and two parameters of annual breeding output.

We always included the year as a random effect, and we removed it from the model formula if the effect on estimate and C.I. calculation was negligible (variance was lower than 0.05 and estimates were the same with and without random factor). Prior to analysis, we standardized (centred and scaled) the continuous predictors to mean zero and units of standard deviation (i.e., z-scores) to ensure comparability among variables, and effects within and across models. Also, we inspected GLMMs and LMMs residuals and considered the dispersion of the data^58^ using a simulation-based approach to create readily interpretable scaled (quantile) residuals for fitted (G)LMMs with the package DHARMa^59^. Model fitting and multi-model inference were carried out using the packages glmmTMB^60^ and MuMIn^61^. Processed tables and a R script is available at https://github.com/paolobecciu/prospecting-female-barn-owls. Descriptive statistics is reported as Mean ± SD, unless specified otherwise.

### Ethics declaration

This study meets the legal requirements of capturing, handling, and attaching GPS devices to barn owls in Switzerland. All experimental protocols and methods were carried out in accordance with relevant guidelines and regulations of, and approved by, the Department of the Consumer and Veterinary Affairs, with legal authorizations: VD and FR 2844 and 3213; VD, FR and BE 3213 and 3571 (capture and ringing permissions from the Federal Office for the Environment). All methods are reported in accordance with ARRIVE guidelines.

## Results

The final dataset included 171 female barn owls—starting from 178 we filtered for tracks with a recording period of at least three nights (see Fig. S2). 35% of females (n = 60) never visited another nest site other than their own (Fig. 1D,E), while the remaining 65% (n = 111, min: 50% in 2017, max: 71% in 2016) visited another nest site at least once (see Fig. 1 for a summary of their prospecting behaviour and its variability). In the models regarding female traits as predictors (see below), we excluded spot diameter because is correlated with number of spots (Fig. S1), hence we decided to use only the latter variable as less subjected to measurement error.

### Factors associated with prospecting behaviour

**Figure 2:**
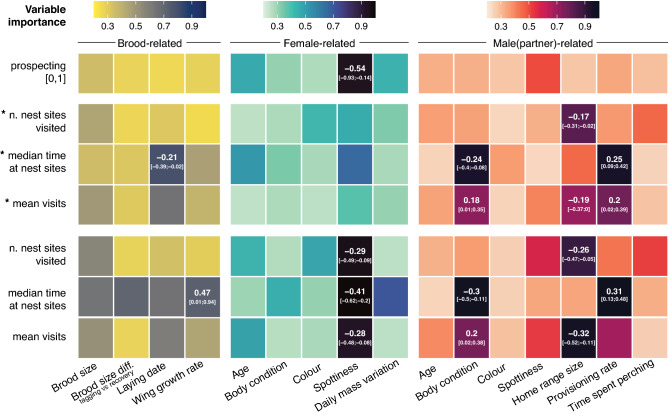
Summary of 21 averaged models (binomial, tweedie and negative binomial GLMMs) displaying the response variables on the y-axis and predictors on the x-axis. The response variables with a preceding “asterisk” include only females that visit other nest sites during their tracking period. Tile colour refers to variable importance derived from model averaging (see “Methods” for detailed explanation). The three different colour palettes highlight the three different set of predictors related with their breeding and brood in yellow to blue, the tracked female in blues, the male partner in red to dark purple. Estimate coefficients and 95% confidence intervals (C.I.) are reported when C.I. do not overlap 0, indicating moderate to strong evidence of the effects reported^62,63^.

In the averaged model series regarding the probability of prospecting or not (prospecting [0,1]) the only variable that had an averaged coefficient with its 95% confidence interval not overlapping with zero was female spottiness (R^2^ = 0.11), while no other variable from models including brood-related (R^2^ = 0.02) and male-related variables (R^2^ = 0.06) was likely associated with the expression of the prospecting behaviour (first row in Fig. 2, Tables S1–S3).

When we considered the continuous response variables excluding the non-prospecting females—*n. nest sites visited, *median time at nest sites, *mean visits—and focussed only on the variation of the behaviour expression, we find several related variables of the male partner as the most important predicting variables as well as the laying date as part of the brood-related factors (Fig. 2, Tables S7–S9).

Number of nest sites visited by female barn owls was negatively associated with male home range size alone (R^2^ = 0.15). Their median time spent at nest sites was associated positively with male provisioning rate, negatively with male body condition (R^2^ = 0.21), and in a weaker way to laying date (R^2^ = 0.06). Females mean visits per nest box were found to be positively related to male provisioning rate, male body condition with moderate evidence of positive effect and negatively related to male home range size (R^2^ = 0.11).

Similar results with slightly higher R^2^ (see Table S6) from male-related variables are shown for the same response variables but including the zeros from the non-prospecting females (n. nest sites visited, median time at nest sites, mean visits; Fig. 2). The main difference between the two sets of models is the strong negative standardised effects of spottiness on all three response variables (Fig. 2, Table S5). Also in this latter case the R^2^ were higher.

### Drivers of multiple visits to a nest box

**Figure 3:**
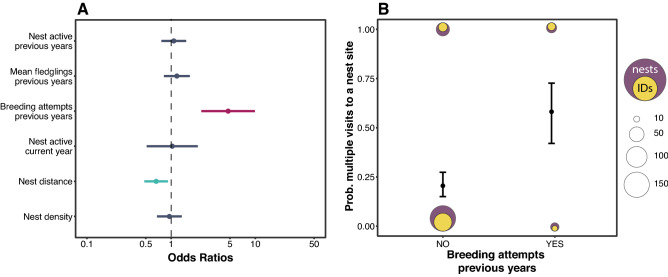
Binomial GLM summary of the results (estimated coefficient with 95% confidence interval ranges) on the probability of revisiting a nest site one or multiple times, dark blue represent standardised effects with 95% C.I. overlapping zero, light purple and light blue represent positive and negative effects, respectively, with 95% C.I. not overlapping zero (**A**). Predicted effect (estimate with 95% confidence intervals) of the predictor with strong evidence of a positive effect given by breeding attempts in the revisited nest in previous years (**B**). Purple dots are nests visited and their size is related to the number of visits per category, yellow dots represent the number of individuals involved.

We tested if a series of parameters concerning nest sites in the area around the current breeding nest of the tracked females could influence their multiple revisitations. The binomial response variable summarises if the nest site is visited only once [0] or more than once [1]. The only factors affecting the probability of multiple visits to a specific nest box were previous nesting attempts by the tracked female in that specific nest box in the previous 15 years, and the distance to that nest box (Fig. 3A, Table S10). Specifically, the probability of a nest to be visited more than once increased from roughly 25% to more than 50%, differing if the nest box visited was never previously used by the tracked female or there was at least one breeding attempt in the 15 years prior (Fig. 3B). In addition, the closer the nest boxes were, the more they were visited by the female owls (Fig. 3A). Other parameters concerning surrounding nest density, such as if the nest was currently occupied or not, how many times was occupied and mean fledged chicks in the previous 15 years were not found to affect the probability of multiple visits (Fig. 3A).

### Mean revisits predict probability of re-nesting

**Figure 4:**
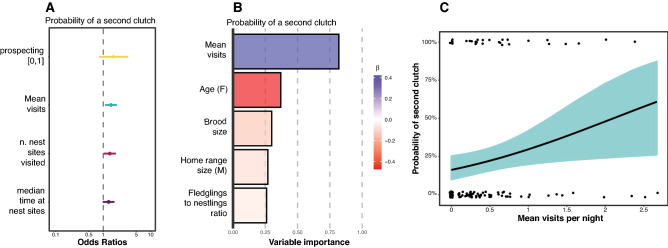
Set of univariate model results (estimated coefficient with 95% confidence interval ranges) with prospecting parameters as predictors of the possibility of having a second clutch by female barn owls, colours are just to visually discriminate the variables (**A**). Summary of variable importance and model coefficients of averaged model (**B**), length of the bar is relative to variable importance (sum of Akaike weights – Σw_i_), colour is relative to the standardised effect direction (red = negative, blue = positive) and its intensity is proportional to the effect strength. Predicted effect of mean visits per night (**C**) from averaged model in (**B**) on the probability of re-nesting for the second time in female barn owls, coloured area around the regression line is relative to 95% confidence intervals and linked to colour of the same variable in (**A**).

We found the mean visits per night was the best prospecting parameter predicting the probability of a second clutch (Fig. 4A) with ΔAIC_c_ > 2 relative to the other univariate models (Table S11). We then included the mean visits per night in a more general binomial GLMM including brood size, age (of the female), home range size (of the male partner) and the proportion of fledglings relative to the number of eggs laid. From the global model we excluded the laying date since it is known to have the largest effect on re-nesting in this species (see^41^), and we confirm this finding in the Supplementary Material (Table S13, Fig. S3). Our model showed moderate evidence of mean visits per night (Fig. 4B, Table S12) with a positive predicting relative contribution on the probability of starting a second clutch (Fig. 4C).

### Implications on annual reproductive fitness

**Figure 5:**
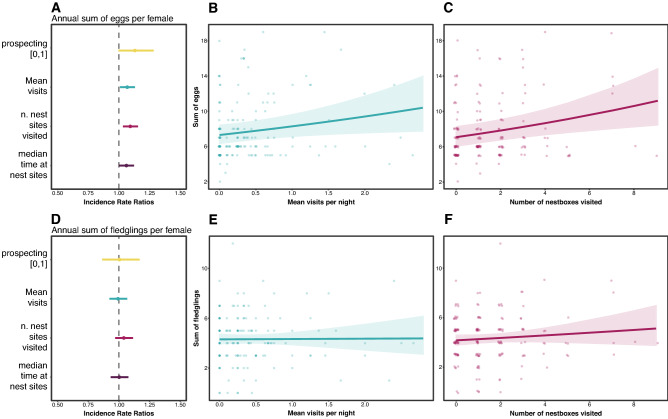
Set of univariate GLMM results (estimated coefficient with 95% confidence interval ranges) with prospecting parameters as predictors of annual sum of eggs (**A**) and annual sum of fledglings (**D**) produced by female barn owls. (**B,C,E,F**) Panels represent the relationship (regression line and 95% confidence interval area) between two prospecting parameters and the annual sum of eggs and fledglings. Colours are different for each prospecting variable and used to link the scatterplots to the effect plots.

As previously described, we ran a univariate model comparison between the prospecting parameters predicting female annual fecundity (sum of eggs per year) and female annual productivity (sum of fledglings per year). For the models with annual eggs as a response variable, the model comparisons highlighted the number of nest sites visited as the best predictor (AIC_c_ = 885.83, β = 0.084, U.C.I. = 0.139, L.C.I. = 0.028), followed by mean visits per night (AIC_c_ = 889.68, β = 0.061, U.C.I. = 0.118, L.C.I. = 0.005), the median time at nest sites (AIC_c_ = 890.06, β = 0.058, U.C.I. = 0.115, L.C.I. = 0.003) and the prospecting[0,1] (AIC_c_ = 891.23, β = 0.107, U.C.I. = 0.231, L.C.I. = −0.016) (Fig. 5A, Table S14). We found that a female expressing the prospecting behaviour was more likely to have more eggs laid during that breeding season (Fig. 5B,C). On the contrary, no prospecting parameter seemed predict the sum of fledglings (Fig. 5D–F, Table S15).

## Discussion

In this study, we described, quantified, and linked a previously unknown prospecting behaviour to possible causes and fitness consequences in breeding females of a nocturnal cosmopolitan predator, the barn owl. Prospecting for breeding sites is known to be a signal of breeding motivation that can be either triggered by external pressures, experience, or genetic predisposition^64,65^. The variation linked to this strategy could also involve and affect parental investment and extra-pair activities^41^, which we do not discuss in depth here. Also, we cannot exclude that female barn owls recorded as non-prospecting during the tracking period, did in fact prospect at other times. The emergence of female prospecting behaviour was found to be related to reduced expression of eumelanin-based colouration (number of dark spots). In vertebrates, melanin-based coloration is often associated with variation in physiological and behavioural traits that probably stems from pleiotropic effects of genes regulating the synthesis of brown to black eumelanin^47^. Heavily spotted barn owl females were found to be more resistant to ectoparasites^66^, to breed at earlier age^48^, to be docile depending on their body condition^67^ and preferable to males that had also a higher feeding rate^68^. Therefore, our finding regarding heavily spotted females being less explorative meaning less time invested in extra-clutch activities (i.e., prospecting for other nest boxes) appears in line with previous studies. Furthermore, these non-prospecting females had a similar annual reproductive fitness than prospecting ones, although the latter laid more eggs (increasing fecundity). A recent study found that barn owl fledglings with larger feather black spots reached farther distances when dispersing from their natal nest to their first breeding nest site^69^. This trait seems related with movement even at an early stage of barn owl life. Although this finding seems to contrast what we found, we should consider dispersal and prospecting while breeding as separate movement behaviours, since we cannot exclude that melanic females disperse more at an early stage of life (before their first breeding) and then do not prospect further during their following breeding seasons, and vice versa for less-melanic females. When we excluded the non-prospecting individuals, the resulting smaller variation in spottiness among prospecting females did not emerge as a strong predictor of prospecting variables. This finding emphasized the role of partner behaviour likely associated to the prospecting behaviour of the females as a stronger and consistent predictor across models compared to spottiness.

Our results showed that variation of prospecting movement among females is likely influenced by their partner behaviour. Male provisioning rate positively affects the time spent and the mean visits per night at visited nest boxes, apparently allowing the female partner to wander more and be less tied to parental duties. On the contrary, smaller male home ranges triggered higher prospecting in terms of nests visited and mean visits per night. Home range size is probably related with higher quality areas and males having higher chances to raise more fledglings^9^. Finally, we found a contrasting effect of male body condition, which had a stronger negative effect on time spent at other nest sites by prospecting females and a moderate positive effect on mean visits per night. On one hand, the first effect indicates that the more the female dedicates time to prospect, the more parental effort is expressed by the male that then pays the price with his body condition. The latter result, on the other hand, shows males in better condition to be associated with higher mean visits per night, although the effect is very weak.

Barn owls are a socially monogamous species and exhibit typical patterns of sexual conflict, as males and females have different parental duties. While males provide most of the prey, females distribute the food to the chicks at the nest. After approximately 20–25 days, females may change their behaviour and either help their partner in providing food or engage in prospecting, resting, and feeding^40^. This shift in behaviour can potentially create a conflict, as the female could use this opportunity to mate with another male and start a second clutch, which would reduce the parental investment in the current brood. However, in our study, we did not observe any evidence of divorce or desertion among prospecting females, resulting in a balanced annual fitness between the sexes. Future research may focus on understanding the role of partner cooperation and coordination in relation to individual and pair fitness outcomes, to quantify the effects of partner choice, parental investment, fidelity, and behavioural coordination in chick provisioning^70^. Importantly, we show an association between male-associated behaviours and their partner’s behaviour, which is one of very few examples in the wider literature where individual movement-based behaviour has been shown to affect that of the respective partner^71^. In the future, we expect that with a growing collection of high-resolution biologging data, in avian and non-avian species, as well as an increasing theoretical and statistical framework on intra- and interspecific interactions^4,5,21,72^ these findings will be more common.

Visiting a particular nest box could highlight a suitability check of the nesting area, and preference is shown if the nest box is already known. We found that more visits were paid to nest boxes that were used by the prospecting female in previous years. This showed an inclination for known and probably suitable territories. This did not mean that in the end the prospecting female succeeded in re-nesting (laying eggs for a second time in the season), and moreover doing it in the preferred nest site. The propensity of re-nesting after the first time in the season greatly depended on when the first clutch was started, as shown in the literature^41,73^ and in this study. But we were surprised to see an important contribution of the prospecting behaviour to predict the probability of having a second clutch. Furthermore, this linked the prospecting behaviour to the annual fecundity of the females, in that more prospecting predicted second clutches and consequently more eggs laid per year. But also to the productivity, that for prospecting females did not increase despite having laid more eggs. In another study on jackdaws (*Corvus monedula*) higher prospecting individuals had less fledglings than non-prospecting conspecifics^42^. Our study and the latter indicate that individuals with distinct prospecting behaviours may adopt varied life-history tactics (such as investing in prospecting or investing in parental care). The advantages and drawbacks of employing different quantities and sources of information are likely dependent on the birds' state (such as their phenotypic traits or previous/current breeding experience). The diversity of states, in combination with the costs of modifying state variables or the presence of positive feedback loops between state variables and information use, could lead to consistent differences in information utilization^74^. Variation in the state might be maintained by fluctuating selection^75^ (e.g., within the context of optimality arguments at selective equilibrium, sub-optimal behaviours can persist), negative frequency-dependent selection^76^ (e.g., fitness of a phenotype decreases as its frequency in a population increases), or life-history trade-offs^77^. Consequently, individuals displaying distinct phenotypes regarding the utilization of social and environmental cues may attain equivalent fitness levels (albeit through diverse pathways) or may adopt the (life-history) tactic that optimizes their fitness according to their own state. Our study suggests that the strategy utilized by female barn owls during prospecting does not have a discernible positive or negative effect on the annual fledgling production, leading to the persistence of both prospecting and non-prospecting strategies with some degree of variation in the population. This could be explained by the fact that male barn owls are primarily responsible for raising chicks, meaning that female contribution to the fledgling's condition is secondary, and as such, annual fitness outcomes may not be heavily influenced by female prospecting behaviour.

In summary, our study utilized high-resolution biologging data to associate prospecting behaviour in a cosmopolitan nocturnal species with its proximal causes and consequences. We demonstrated (1) the significance of this technology in remotely identifying and quantifying nocturnal behaviours (such as prospecting) and linking them with landscape features, (2) the connection between the emergence and variability of prospecting behaviour and phenotypic- and fitness-related variables, including movement-related parameters such as home range and activity budget, and (3) the relationship between prospecting behaviour and female annual fitness outcomes, highlighting sex-specific breeding strategies in a wild barn owl population. Overall, our study highlights the valuable contributions of biologging technology and field monitoring to the fields of movement and behavioural ecology.

## Supplementary Information

Supplementary Information.

## Data Availability

The GPS datasets analysed in the current study are available in Movebank (www.movebank.org), under the project named “Barn owl (*Tyto alba*)”, ID 231741797 (https://www.movebank.org/cms/webapp?gwt_fragment=page=studies,path=study231741797). Processed tables and a R script are available at: https://github.com/paolobecciu/prospecting-female-barn-owls.
